# Study protocol: the Norwegian Triple-S Cohort Study - establishing a longitudinal health survey of children and adolescents with experiences of maltreatment

**DOI:** 10.1186/s12889-021-11125-9

**Published:** 2021-06-05

**Authors:** Viktor Schønning, Anders Dovran, Mari Hysing, Gertrud Sofie Hafstad, Kristin Stokke, Leif Edvard Aarø, Stian Tobiassen, John Are Bjerge Jonassen, Øystein Vedaa, Børge Sivertsen

**Affiliations:** 1 Department of Health Promotion, Norwegian Institute of Public Health, Postboks 973 Sentrum, 5808 Bergen, Norway; 2 Department of Psychosocial Science, University of Bergen, Bergen, Norway; 3 Stine Sofie Foundation & Stine Sofie Centre, Grimstad, Norway; 4 Department of Psychosocial Health, University of Agder, Grimstad, Norway; 5 Norwegian Centre for Violence and Traumatic Stress Studies, Oslo, Norway; 6 Department of Mental Health, Norwegian University of Science and Technology, Trondheim, Norway; 7 Voss District Psychiatric Hospital, NKS Bjørkeli, Voss, Norway; 8 Department of Research and Development, St Olavs University Hospital, Trondheim, Norway; 9 Department of Research & Innovation, Helse Fonna HF, Haugesund, Norway

**Keywords:** Protocol, Childhood maltreatment, Adverse childhood experiences, Abuse, Adolescence, Longitudinal, Epidemiology, Mental health

## Abstract

**Background:**

Child maltreatment is prevalent and associated with both short- and long-term health problems. Previous studies have established child maltreatment as a risk factor for a wide range of problems over the life course such as mental- and somatic health problems, self-harm, alcohol- and drug abuse and decreased work-life participation. Still, there are few large and well-conducted longitudinal studies focusing on describing prevalence and identifying risk factors and long-term consequences of child maltreatment. The purpose of the current study is to recruit a large number of children and adolescents exposed to maltreatment and follow them long-term.

**Methods/design:**

The current study is a longitudinal cohort study and will use a multi-informant design (child/adolescent, caregiver, and administrative data). Participants will be recruited from the Stine Sofie Centre (SSC), a learning and coping centre for children and adolescents (≤18 years) exposed to maltreatment, which includes physical and emotional abuse, neglect and/or sexual abuse. Questionnaire-based assessments from self-reports (as well as parent-reports) will be carried out at regular time intervals throughout their lives, on topics such as abuse, negative life events, mental and somatic health problems, resilience and coping, satisfaction with health services, social-, family-, and school function, as well as self-harm and substance abuse. Participants will be assessed upon entry to the centre and followed up annually until they reach 18 years and bi-annually after. Given written consent, participants’ responses will be linked to relevant national registries in order to examine predictive factors and important outcomes in terms of subsequent health, education, criminal records and work affiliation.

**Discussion:**

This study will examine short- and long-term consequences of child maltreatment across a range of health-related outcomes in a longitudinal perspective. Results from the current study might have implications for the development of preventive and intervention programs related to child maltreatment and the organization and follow-up of the services these children receive. The current study will hopefully contribute with knowledge of risk-factors, short- and long-term health-related and other issues that can contribute to practices aimed at improving the overall life-course for children and adolescents who have experienced childhood maltreatment.

## Background

Child maltreatment is a global problem and associated with major long-term consequences. Child maltreatment includes all types of physical and/or emotional ill-treatment, sexual abuse and neglect which occur in the context of a relationship of responsibility, trust or power, and which result in actual or potential harm to children’s health, survival, development or dignity [1]. Exposure to childhood maltreatment is common, with prevalence rates varying across the different types of maltreatment [2, 3]. A review from 2009 estimated that in industrialized countries, 4–16% of children had been physically abused, 10% had been psychologically abused, while 5–10% had been sexually abused [4]. A review of a series of meta-analyses on global child maltreatment estimated prevalence rates of 13% for sexual abuse, 23% for physical abuse, 36% for emotional abuse, 16% for physical neglect and 18% for emotional neglect from self-report studies assessing maltreatment during childhood [5]. In contrast, a recent Norwegian study of the general adolescent population found that 19% had experienced some form of physical abuse (including mild and severe violence), 6% had experienced sexual abuse from adults, while 22% had experienced some form of sexual abuse from peers [6].

Childhood maltreatment is associated with several adverse and potential life-long health consequences [7–10]. Children and adolescents exposed to maltreatment have increased risk of later mental and somatic health problems and disorders [9, 11–13] and subsequent revictimization later in life [14]. Exposure to one type of maltreatment also increases the risk of polyvictimization [2, 14–17]. However, as emphasized by Kessler et al. [9], there is a lack of studies exploring potential mechanisms that could help explain these associations, and they advised that future studies should explore and identify mediators, modifiers and developmental sequences that might be of importance. A similar conclusion was drawn in a prospective study, where children with a history of sexual abuse developed more externalizing and internalizing problems in adolescence [18]. The authors of that study also emphasized the need for future studies to examine both mechanisms and possible gender differences in this age cohort [18].

Lower socioeconomic status (SES) is associated with both increased exposure and reduced resources to cope with the stressors [19], and higher SES has been found to have a protective effect on the health outcomes of childhood maltreatment [20]. However, the association between SES and childhood maltreatment is complex, and more research has been called for to further disentangle the mechanisms involved in this association [20].

Given that childhood maltreatment is believed to be among the most potent risk factors for poor health and reduced daily functioning [4], there are surprisingly few large and well-conducted studies focusing on identifying both risk factors, and long-term consequences of the specific types of childhood maltreatment. This leaves an inadequate knowledge base for developing effective interventions. A meta-analysis from 2017 stated that future studies would benefit from designs that allow stronger causal inference and control for factors that attenuate or amplify observed relations [21]. The traditional means of data collection to examine these issues are population-based health surveys, which are well-suited to establish prevalence estimates but rarely offer the opportunity to include many participants exposed to childhood maltreatment. Few studies to date have recruited a large number of children and adolescents with experiences of childhood maltreatment and followed them long-term, while identifying risk factors and subsequent health outcomes. Detailed knowledge of specific mechanisms and developmental trajectories of children exposed to maltreatment is pivotal to improve prevention of child maltreatment. The World Health Organization recently identified several gaps in this research field [22], highlighting the need to identify both risk- and protective factors, help-seeking behaviors, long-term impact, and nuanced knowledge of different service needs across gender, ethnicity, sexual orientation, disability and socioeconomic status [22]. The importance of studies with longitudinal and prospective research designs, and the importance of accounting for pre-existing vulnerabilities has also been emphasized as a future research need [23].

### Aim of the project

The main aim of the project is to establish a longitudinal health cohort of children and adolescents with experiences of maltreatment, as well as their close family members. The abbreviation “Triple-S” originates from the Norwegian name of the Stine Sofie Centre, “Stine Sofie senteret”. The goal of the Norwegian Triple-S Cohort Study (Triple-S study) is to carry out data collections annually/biannually from children with experiences of maltreatment and their families (i.e., multi-informant design), and subsequently to link this information to official data from national registries on health, education, and work affiliation (among others). This will provide new knowledge on predictors and short- and long-term consequences of childhood maltreatment, and contribute to providing a scientific basis for policymaking, program planning and targeting service delivery. The project will also aim to identify mediators and modifiers to help explain the documented associations between childhood maltreatment and health-outcomes and compare to other relevant populations.

## Methods/design

A longitudinal multi-informant cohort study on children and adolescents exposed to maltreatment, as well as their parents or legal guardian.

### Participants

The SSC is run by The Stine Sofie Foundation (SSF) and was founded in 2000 after the Baneheia murders where 10-year-old Lena Sløgedal Paulsen and 8-year-old Stine Sofie Sørstrønen were raped and murdered. The SSF has since its establishment worked to strengthen the legal security of maltreated children/adolescents and their relatives, and for the prevention of child maltreatment. In 2016 the SSF established one of the world’s first learning and coping centres for children and adolescents exposed to physical and emotional abuse, neglect and/or sexual abuse, the SSC, with financial support from the Norwegian government [24]. Children or adolescents who have been victims of either physical or psychological violence, sexual abuse or who have witnessed domestic violence – and have this confirmed by their primary physician, Child Welfare Services or other officials – are eligible course participants at the SSC. The children/families must complete an application for a place at the SSC before they are admitted as course participants.

The main aim of a stay at the centre is to help the child or adolescent with the recovery process from their maltreatment experience. This is done by strengthening the child’s resilience to stress, where learning, coping-focused activities and positive experiences are pivotal. Children and adolescents who attend the centre receive ability- and age-appropriate teaching about brain functioning, ways of reacting and different techniques for emotional regulation. Throughout the stay, the participants are given the opportunity to challenge themselves both physically and creatively through tailored activities. The teaching takes place in small, short sequences, where visual aids such as videos, music, relaxation exercises, plays and outdoor excursions are used to reinforce the experience of learning in a non-judgmental and safe environment. The program is playful and positively charged, with a focus on coping-ability. A fundamental element is that all teaching and other activities at the SSC should be transferable to the home situation and help the children handle future difficulties. Currently, very few similar public or private services that cover all the needs demanded by this cohort exists. This is in sharp contrast to both the high incidence of violence and abuse against children, as well as the extent of the well-documented harmful effects.

The number of children and adolescents visiting the SSC has steadily increased over the last few years, and in 2019 more than 500 children and their caregivers stayed at the centre. It is estimated that this annual figure will continue to increase to approximately 700 children and their caregivers per year. As such, we aim to include around 2500 children and their caregivers for the present study during the recruitment phase. The recruitment phase commenced in January 2021. The number of families staying at the SSC has been downscaled because of restrictions related to the COVID-19 pandemic. As a result of this, the recruitment phase will be extended until we reach our aim of 2500 children and caregivers. The age distribution of participants at the SSC in 2016–2020 was 30% from 0 to 7 years, 40% from 8 to 11 years and 30% from 12 to 18 years. We estimate that the age distribution of recruited participants will be similar to prior years.

The SSC has a follow-up team which provides support and help for all former participants at the SSC. During the last 2 years, the follow-up team has supported over 50% of the participating families on the families’ request. There is also an app named “My Stay” where the participants’ can access course materials from the SSC.

### Recruitment

All children and adolescents aged 5 to 18 years and their caregivers that have been approved for a stay at the SSC will be invited to participate in the study. Each potential participant will be personally invited when they are admitted as course participants prior to their arrival at the SSC. When the children and caregivers arrive at the SSC, age-appropriate written information about the project will be given by personnel at the SSC. Researchers and health personnel are available for further questions regarding participation. To avoid a feeling of coercion, children and families at the SSC are informed that participation in the study does not affect their stay at the SSC.

### Data collected and instruments used

The current study is a longitudinal cohort study and will use a multi-informant design (child/adolescent, caregivers, and administrative information). We will cover several domains, including mental and physical health, resilience, adverse life events, and functioning (social, family, school, etc.). Administrative data on referral information and background information will also be collected. The first data collection will be conducted using tablets on a secure web-based platform during the participants’ stay at the SSC. Subsequent data collections will be conducted annually until the participants turn 18 years, and then bi-annually, using the same secure web-based platform. Table 1 provides details of the planned assessments. The assessment batteries will include a combination of both questionnaires used in other large Norwegian epidemiological surveys (e.g. the Norwegian mother and child cohort study [46], the Bergen Child Study [47], and the Norwegian youth study on child maltreatment (the UEVO study) [6]) to enable comparisons on key variables with age-matched peers, as well as questionnaires specifically relevant for this particular cohort. Data from the three mentioned epidemiological surveys will be used for comparisons on selected variables.

**Table 1:** Overview of the main included instruments/variables in the Triple-S study

Domain	Instruments and description	Caregiver 5–11 years	Self-report & parent-report 12–15 years	Self-report & parent-report 16–18 years	Self-report caregivers	Administrative
Maltreatment	Traumatic events and post-traumatic stress responses is assessed by Child and Adolescent Trauma Screen (CATS) [25].	X	X	X		
	Childhood Trauma Questionnaire (CTQ) [26] is used to assess traumatic experiences in childhood.				X	
	Neglect and psychological abuse is assessed using questions from the Juvenile Victimization Questionnaire [27] formerly used by Jernbro & Janson [28].	X	X	X		
	Questions used by Jernbro & Janson [28] will assess physical abuse, witness to partner violence, online sexual abuse, sexual abuse from adults and sexual abuse from peers.	X	X	X	X	
	Reasons abuse was discovered, and reasons the child/adolescent did not come forward with the abuse (if they did not).	X	X	X		
	Knowledge of peers’ experience with abuse		X	X		
	Reason for referral					X
Physical health	Physical health is assessed a pre-defined list of physical, mental and developmental conditions. The list covers the most frequent conditions and is similar to lists used in other large population studies such as the HUNT study [29].	X	X	X	X	
Pain	Somatic health/pain is assessed by the Somatic Symptom Scale-8 (SSS-8) [30] which is an 8-item self-report measure of somatic burden.		X	X	X	
Sleep	Sleep is assessed by sleep duration variables, nightmares and insomnia by DSM-5 criteria [31].	X	X	X	X	
Mental health and well-being	Psychological distress is assessed using The Hopkins Symptom Checklist (HSCL-14) [32], a screening tool to detect anxiety and depression symptoms derived from the 90-item Symptom Checklist (SCL-90).				X	
	The Strength and Difficulties Questionnaire (SDQ) is used to assess emotional and behavioural problems [33].	X	X	X		
	Externalising disorders is assessed using the Parent/Teacher Rating Scale for Disruptive Behaviour Disorders [34].	X				
	Aggression is assessed by The Appetitive Aggression Scale (AAS) [35]		X			
	Conduct problems is assessed by The Disc Predictive Scales [36]			X		
	Satisfaction With Life Scale is used to measure well-being through 5 items [37].				X	
	Short Mood and Feelings Questionnaire [38] is used to measure depressive symptoms.	X	X	X		
	Self-harm and suicidality is measured by CASE [39].	X	X	X	X	
	Resilience is measured by the Resilience Scale for Adolescents [40].		X	X		
ADHD	ADHD symptoms is assessed using the Swanson, Nolan and Pelham Questionnaire (SNAP-IV) [41].	X		X		
	ADHD symptoms is assessed using the Adult ADHD Self Report Scale (ASRS) [42].			X		
Help-seeking behaviour	Help-seeking behavior is assessed by history and satisfaction with contact with health-care institutions. If they have not received help, they are asked the reasons why.	X	X	X		
Health behaviours	Physical activity and sedentary behavior is assessed through questions of frequency and quantity of activity.	X	X	X		
	Social relations is assessed using “The Three Item Loneliness Scale” [43] and number of close friends.		X	X		
	Smoking and use of snus (tobacco) will be assessed by measures of frequency and quantity of use.		X	X	X	
	Alcohol use is assessed by measures of frequency and quantity and the CRAFFT screening test [44].		X	X	X	
	Drug use is assessed measures of frequency and quantity of use.		X	X	X	
Background	Living situation, family constellation and upbringing.	X	X	X		
	Education, work affiliation and income				X	
Other information	Parenting style is assessed by parts of the Alabama Parenting Questionnaire [45].	X	X	X		
	Relation between caregivers is assessed by questions about parenting styles used in studies on similar age-groups.				X	
	School functioning will be assessed through absence, grades and how they feel about school.	X	X	X		

#### Study measures

Table 1 outlines an overview of key instruments to be included in the project. Note that this is not an exhaustive list of all included questionnaires and items. The participants will receive age-appropriate questionnaires, with adolescents from 12 to 18 years providing information through self-report and caregivers providing information on children from 5 to 11 years. Caregivers for adolescents from 12 to 18 years will provide information on their own mental and physical health, life events, drug use and functioning, in addition to some parent-reported information on the adolescents. All caregivers will provide information of themselves.

Our aim is to include collection of biological material at a later time, to allow the examination of genetic work-up and neurobiology markers. At the current moment we do not have sufficient funding or ethical clearance to include these analyses.

#### Linkage to national registries

The project will also allow linkages to several national registries in order to examine both predictive factors as well as to investigate important outcomes such as subsequent health, education, and work affiliation. The linkages to the national registries have been approved by the Regional Committees for Medical and Health Research Ethics. We have also conducted a Data Protection Impact Assessment which describes the handling of personal information. Table 2 provides an overview of planned linkages to possible registries.

**Table 2:** Overview of other data sources/registers scheduled to be linked to the health survey, given additional funding

The Medical Birth Registry of Norway (MBRN)	MBRN is a national health registry containing information about all births in Norway. The registry has been widely used to identify causes and consequences of health problems related to pregnancy and birth. The MBRN also includes information about maternal health before and during pregnancy.
The KUHR/KPR database	The KUHR database is owned by the Norwegian Directorate of Health, and includes data on reimbursement to GPs for the health care service they provided to primary health care service users. The report sent by each GP contains detailed information about the diagnosis and treatment.
The Norwegian Prescription Database (NorPD)	The NorPD monitors drugs dispensed by prescription in Norway, and contains data on all prescriptions, including type of medication (ATC-code), and dosage. All pharmacies in Norway register prescriptions electronically, and the information is sent in monthly reports to NorPD.
The Norwegian Patient Registry (NPR)	The NPR is a comprehensive registry of inpatient and outpatient hospital care in Norway. The registry contains detailed data on each individual’s history of diagnose(s) and treatments from the high school years throughout his/her college/university education.
The National Educational Database (NUDB)	The NUDB includes information about completed education at all levels, grades and school-drop out. The database includes individually based statistics on education since 1970.
The Norwegian Cause of Death Registry	The Norwegian Cause of Death Registry includes information on cause of death for all deceased persons registered as residents in Norway at the time of death.
NAV & FD-Trygd (Norwegian Social Insurance Database)	All social insurance benefits are accurately recorded in the Norwegian Social Insurance Database. The data in the registries includes type of benefit, degree of compensation, start and end date of benefit recipiency and medico-legal diagnosis.
Criminal Record	The criminal record is a nationwide register of punished persons and is kept by The Norwegian Criminal Investigation Service. The register includes information on criminal convictions, penalties and other decisions involving custodial sentences, community punishment, juvenile punishment, penalties for offenses, transfer to compulsory mental health care or forced care etc.

### Project management

The project will be a joint effort between the SSF and the Norwegian Institute of Public Health (NIPH). The initial funding is from the SSF the first 5 years (from Oct 1st, 2019 to Sept 30th, 2024), and we will apply for additional funding to ensure the continued activity of the project. The NIPH will be the responsible research institution and will collaborate closely with researchers from the SSC, the University of Bergen, the University of Agder, and the Norwegian Centre for Violence and Traumatic Stress Studies.

### User involvement

All parts of the project, including the planning of research questions, selection of questionnaires, collection of data, as well as utilization of data and findings, are conducted in close collaboration with the Triple-S study steering committee. The project group will routinely meet with the Triple-S study steering committee to discuss relevant aspects of this research. To ensure user involvement, a representative from the National Association for Child Welfare-Children will serve as an active member of the external work group. This is a user organization, and the representative has participated in a project seminar and provided feedback and input on study design and choice of questionnaires. Multilayered user involvement will be ensured by inviting representatives from involved families/project participants to attend project seminars. Prior to commencing data collection, we conducted pilot-tests of the questionnaires, followed by focus-group discussions among user groups of children, adolescents and caregivers. In the focus group discussions, participants’ gave feedback on study design, choice of questionnaire instruments and scales, and practical solutions which led to several changes in important aspects of the study. Furthermore, in this research project, it is seen as important that the child rights perspective is recognized, and the users involved at all levels and in all stages.

### Ethics

The study protocol has been approved by the Regional Committee for Ethics in Medical and Health Research in the south-eastern region of Norway (#95445). If participants wish to participate, children and adolescents from 12 to 18 years will be invited to provide an electronic consent. In Norway, adolescents from 12 years of age are allowed to independently provide a written consent to participation in health research when the topic of research is abuse, domestic violence or other issues where there might be a conflict of interest between child and parent, and the parent might have an interest in denying their child to participate in the study. For children between 5 and 11 years, legal guardians (parent, foster parent or other) will provide written consent as required by law. Adult caregivers will provide written consent on their own behalf. Participants must provide their unique national personal identification number when consenting to participate. The consent form includes two separate voluntary consents: 1) participation in the main survey, and 2) for linking their data from the survey to external registries.

Project management will receive the written consent directly without passing through health personnel at the SSC. For participants that have experienced maltreatment, questions on these sensitive subjects might be uncomfortable and difficult to answer. The current questionnaire can potentially be experienced as burdensome for the participants as the questions include personal subjects such as abuse, self-harm and mental health. We have conducted a pilot of the questionnaire with participants at the SSC and the overall feedback was that the sensitive nature of the questions were not experienced as particularly bothersome. The time to complete the questionnaire varied from 20 to 55 min across the age groups. To ensure adequate care for participants during the data collection, personnel from the SSC will be present during the first data collection and available via telephone during all later data collections to participants in need of support or debriefing. All collected data will be handled according to the newly adopted General Data Protection Regulation.

Participants’ are informed that personnel at the SSC do not have the possibility to view their questionnaire data, and if they experience any immediate issues that will require involvement, they will have to contact the personnel directly. If any criminal incidents are discovered at the SSC, the relevant authorities will be contacted immediately according to national laws.

### Statistical analysis

In addition to the specific focus of the potential individual studies, the design permits a comprehensive exploration of many critical issues in the field of child maltreatment on a sample comprising both children and parents of sufficient size for the most central purposes. Dimensionality and psychometric properties (including internal consistency) of scales will be examined. Depending on the purpose, sum scores or mean scores will be constructed or we will use latent variable approaches. In both of these cases, various forms of structural equation modeling will be applied. Since the project is based on a prospective longitudinal design (panel data), we will also use statistical tools such as growth curve modeling and state of the art approaches to cross-lagged analyses. This will allow us to investigate on possible causal processes. For handling data, for descriptive (frequency tables, means) and for statistical analyses like exploratory factor analysis, GLM and logistic regression analyses, STATA will be applied. When using structural equation modeling with latent variables we will use Mplus in addition to R-based Lavaan. Other statistical tools will be applied when deemed adequate. We have planned comparative data collections, in order to examine differences between our study population that has been subjected to maltreatment and general population samples across a wide range of health-related outcomes.

### Statistical power

We aim to recruit participants over at least a 5-year time period, and based on the number of annual participants at the SSC (currently approx. 700) we aim to recruit 2500 children and adolescents. This number is estimated to be adequate to examine our main research questions. Providing statistical power analyses for each of the large number of possible research questions in this study is not possible. However, some indication of the level of statistical power can be provided. With a sample size as large as 2500 a 95% confidence interval (C.I.) for a 50% proportion is +/− 1.98% and of course smaller for proportions smaller or larger than 50%. As the current study population is highly selected, we expect most of the phenomena we wish to study to be sufficiently prevalent. When estimating proportions for subgroups as small as 10% of the sample (*n* = 250), the 95% C.I. for a 50% proportion is +/− 6.37%. The corresponding C.I.s for the mean of a metric variable are +/− 0.04 standard deviations (*n* = 2500) and 0.12 standard deviations (*n* = 250). Phrased slightly differently; with a significance level of 5%, statistical power of .80 and testing a 10-percentage points difference between two proportions (55% versus 45%), the number of observations needed in each group is 392. In the context of latent variables and structural equation modeling, and even multiple linear regression analysis, samples as large as 1000 to 2500 are considered more than sufficient for most purposes. In a multiple linear regression analysis with 8 predictors, R square equal to 0.10, significance level of .05 and a sample size of only 150, the statistical power is as high as .82. In a study as broad and multi-faceted as the present one, new hypotheses and models to be examined will develop as the study unfolds. The larger sample, the more complex models involving various combinations of predictors, covariates, mediators and moderators can be examined, and the smaller subgroups can be subject to statistical analyses. An even larger sample could have permitted testing of more complex models and provided opportunities to examine even smaller subgroups. Availability of participants and time constraints prevent further expansion of the study sample.

## Discussion

With the current study we aim to gain knowledge and insight into the consequences of child maltreatment on a wide range of health-related outcomes. The longitudinal, multi-informant design and linkage to national registries allows examination of a wide range of research questions and relationships among variables. We will assess caregivers’ health and maltreatment history, and thus examine inter-generational factors possibly contributing to childhood maltreatment, and children and adolescents’ subsequent health. Experiencing childhood maltreatment has been found to be a robust predictor of subsequent revictimization, and the linkage to national registries and long-term follow up allows us to examine the frequency and severity of revictimization for individuals exposed to maltreatment in childhood [14]. An important issue for studies focusing on childhood maltreatment is being able to separate different kinds of maltreatment and their associated outcomes. Poly-victimization is common for this population, and there may be some challenges in separating which outcomes are related to which type of abuse in the situations where a participant has experienced poly-victimization. To meet this challenge we will analyze exposure to maltreatment using a cumulative and a dimensional approach [48]. We have also included a detailed assessment of the different kinds of maltreatment, including a timeline of the type of abuse, the number of times the abuse has happened and the participants’ relation to the abuser. The questionnaire assesses physical-, sexual-, and emotional abuse, physical- and emotional neglect and witness to violence.

Identifying mediators, moderators and important developmental sequences is a focus in the current study, to be able to explain the documented associations between childhood maltreatment and later unfavorable health outcomes [9]. Some of the moderators we will examine are socio-economic status, gender, social support and follow-up by health care personnel, including the timing of receiving health care. These moderators are likely to be of importance, and have been found to moderate the association between maltreatment and negative health consequences [49]. The mediators of interests will be specific to the research question of the specific study. However, some prior research has identified life satisfaction [50], interpersonal difficulties [51] and coping mechanisms [51] as possible mediators of the association between maltreatment and later depressive symptoms and emotional distress. These are examples of potential mediators we assess in the current study and have the possibility to include in sub-projects. We also wish to study the importance of developmental sequences and examine how children are affected by maltreatment at an early age (5–11 years) compared to during puberty or late-adolescence.

Even though the participants’ stay at the SSC is not considered treatment, they may receive a boost to resilience during the visit and no longer be completely representative of most maltreated children. Any improvement to participants’ resilience will most likely not have occurred at the time of the baseline data collection. Still, we cannot disregard the possibility that some participants’ may experience long-term improvements in their resilience as a result of their stay at the SSC. To control for this possibility, we will compare our findings with similar data from three other studies, including the UEVO-study [6] which recruited 9240 adolescents from the general population, assessing maltreatment history in detail. The UEVO-study will provide important comparative data to help illuminate any differences between the general population and participants at the SSC.

Hopefully, the Triple-S study will help shed light on some of the unmet needs of children and adolescents experiencing maltreatment. Our aim is that results from the current project will help improve prevention of childhood abuse, treatment and overall life-course for children and adolescents who experience childhood maltreatment.

### Limitations

There are some challenges to the current study. Response rates tend to be low in health surveys, particularly over telephone or mail. As the participants are attending the SSC while answering the initial questionnaire we expect the response rate to be higher than usual for this population [52]. A comparable study, the UEVO study, delivered questionnaires with researchers physically present and obtained a response rate of 86%, which we believe is attainable [6]. The recruitment phase for the Triple-S study started in January 2021, and currently, our response rate is approximately 85%. Another possible limitation is that that children and adolescents who apply to and visit the centre might represent a selected and more resourceful group as compared to the entire population who experience maltreatment. However, 60–70% of the referrals received by the SSC comes from the child protection services and out-patient clinics, suggesting that the population at the SSC represents children and caregivers that really are in need of help and not necessarily more resourceful than others that experience childhood maltreatment. The current study mostly relies on self-report and parent-report data with its inherent limitations. However, we will also utilize supplemental administrative information which can provide more objective data than self-report as well as linkages to more objective registry data. A strength of the study is the planned utilization of registries which will provide antecedent and prospective information that allows long-term follow-up. More objective forms of data collection has been highlighted as needed in the research field of child maltreatment [23]. As in any longitudinal study there is the risk of drop-out. However, former data collections at the SSC suggests that the user group shows commitment to participate in studies on the subject matter. Preferred measures to decrease drop-out in a longitudinal perspective have been discussed through pilot testing and focus-group interviews with the user group.

## Data Availability

The data collected in this study are not publicly available because of privacy regulations from the Norwegian Regional Committees for Medical and Health Research Ethics (REC). Requests to access the datasets should be directed to the NIPH (Datatilgang@fhi.no). Guidelines for access to NIPH data are found at https://www.fhi.no/en/more/access-to-data. Approval from REC (https://helseforskning.etikkom.no) is a pre-requirement.

## Abbreviations

SSC: The Stine Sofie Centre
SSF: The Stine Sofie Foundation
SES: Socioeconomic status
Triple-S study: The Norwegian Triple-S Cohort Study
the UEVO study: The Norwegian youth study on child maltreatment
NIPH: The Norwegian Institute of Public Health
C. I.: Confidence Interval
